# Molecular characterization of echovirus 12 strains isolated from healthy children in China

**DOI:** 10.1038/s41598-018-30250-x

**Published:** 2018-08-06

**Authors:** Hongbo Liu, Jie Zhang, Yilin Zhao, Haihao Zhang, Keqin Lin, Hao Sun, Xiaoqin Huang, Zhaoqing Yang, Shaohui Ma

**Affiliations:** 1 Institute of Medical Biology, Chinese Academy of Medical Sciences and Peking Union Medical College, Kunming, 650118 PR China; 2 Yunnan Key Laboratory of Vaccine Research Development on Severe Infectious Disease, Kunming, 650118 PR China

## Abstract

Human echovirus 12 (E-12) belongs to the enterovirus B species. To date, only one full-length genome sequence of E-12 (prototype strain Travis) is available in the GenBank database. This study determined the complete sequence of three E-12 strains, which were isolated from the stools of three healthy children in Yunnan, China, in 2013. We revealed that the three Yunnan E-12 strains had only 80.8–80.9% nucleotide identity and 96.4–96.8% amino acid identity with the Travis strain based on pairwise comparisons of the complete genome nucleotide and amino acid sequences. The three Yunnan strains shared 99.7% nucleotide identity and 99.1–99.5% amino acid similarity. Phylogenetic and similarity plot analyses showed that intertypic recombination occurred in the non-structural regions of the three Yunnan E-12 strains. This is the first report of the complete genome sequence of E-12 in China and it enriches the complete genome sequences of E-12 in the GenBank database.

## Introduction

Enteroviruses (EVs) are members of the genus *Enterovirus*, family *Picornaviridae*, and are further divided into 12 species: enterovirus A–H, J and rhinovirus A–C^1^, comprising more than 100 serotypes. EVs are single-stranded, positive, non-enveloped small RNA viruses. The genome of EV contains approximately 7,400 nucleotides including a long open reading frame (ORF). It encodes a polyprotein that is cleaved into 4 structural proteins (*VP4*, *VP2*, *VP3*, and *VP1*) and 7 non-structural proteins (*2A*, *2B*, *2C*, *3A*, *3B*, *3C*, and *3D*) flanked by a 5′-untranslated region (UTR) and a 3′-UTR^1,2^. EVs can cause a wide range of diseases, such as fever, hand-foot-and-mouth disease (HFMD), aseptic meningitis, acute flaccid paralysis (AFP), encephalitis, and myocarditis. However, most EV infections are asymptomatic or result in mild symptoms. Enterovirus B (EV-B) species include 6 coxsackievirus B (CV-B) serotypes, coxsackievirus A9 (CV-A9), 28 echovirus (E) serotypes, enterovirus B69 and 25 new types^3^.

Echoviruses are the major cause of aseptic meningitis^4–6^. They share a similar genome structure to other EVs. They are commonly associated with asymptomatic or mild infections but can also cause serious diseases such as AFP^7^. However, E-12 is not a prevalent EV serotype and was previously isolated through AFP surveillance^8^. There is currently only one genome sequence of E-12 in the GenBank database, which was isolated from the intestine of a patient with diarrhoea and aseptic meningitis^9^. In the present study, we report the complete genome sequences of three additional E-12 isolates that were recovered from three healthy children aged 3–6 years in the Yunnan Province of China in 2013. The determination and analysis of the complete genome sequence of E-12 will help to elucidate the origin and evolutionary characteristics of E-12 in China and provide beneficial information for related disease surveillance.

## Results

### Isolation and typing

The eight isolates (K603/YN/CHN/2013, K605/YN/CHN/2013, K606/YN/CHN/2013, K624/YN/CHN/2013, K629/YN/CHN/2013, K630/YN/CHN/2013, K646/YN/CHN/2013 and K1529/YN/CHN/2013) were isolated from KMB17 (human embryonic lung diploid fibroblast) cells. The eight strains were confirmed to be E-12 by the amplification of the partial *VP1* sequences. The sequences were analysed using an online enterovirus genotyping tool. They were isolated from six males and two females with ages ranging from 3 to 6 years.

### VP1 sequence analysis

The complete *VP1* sequences of the eight Yunnan strains had the highest similarity with other E-12 strains in the GenBank database and had a percentage reaching 98%. The eight strains were identified as E-12 using an EV molecular typing criteria.

The eight isolates had 79.7–80.6% nucleotide and 93.8–96.3% amino acid similarity with the complete VP1 coding region of the E-12 prototype strain Travis. They shared 60.7–79.1% nucleotide and 60.5–85.2% amino acid similarity with the prototype of other EVs. The complete *VP1* sequence and amino acid similarities among the eight Yunnan strains were 98.4–100% and 96.6–100%, respectively.

**Figure 1:**
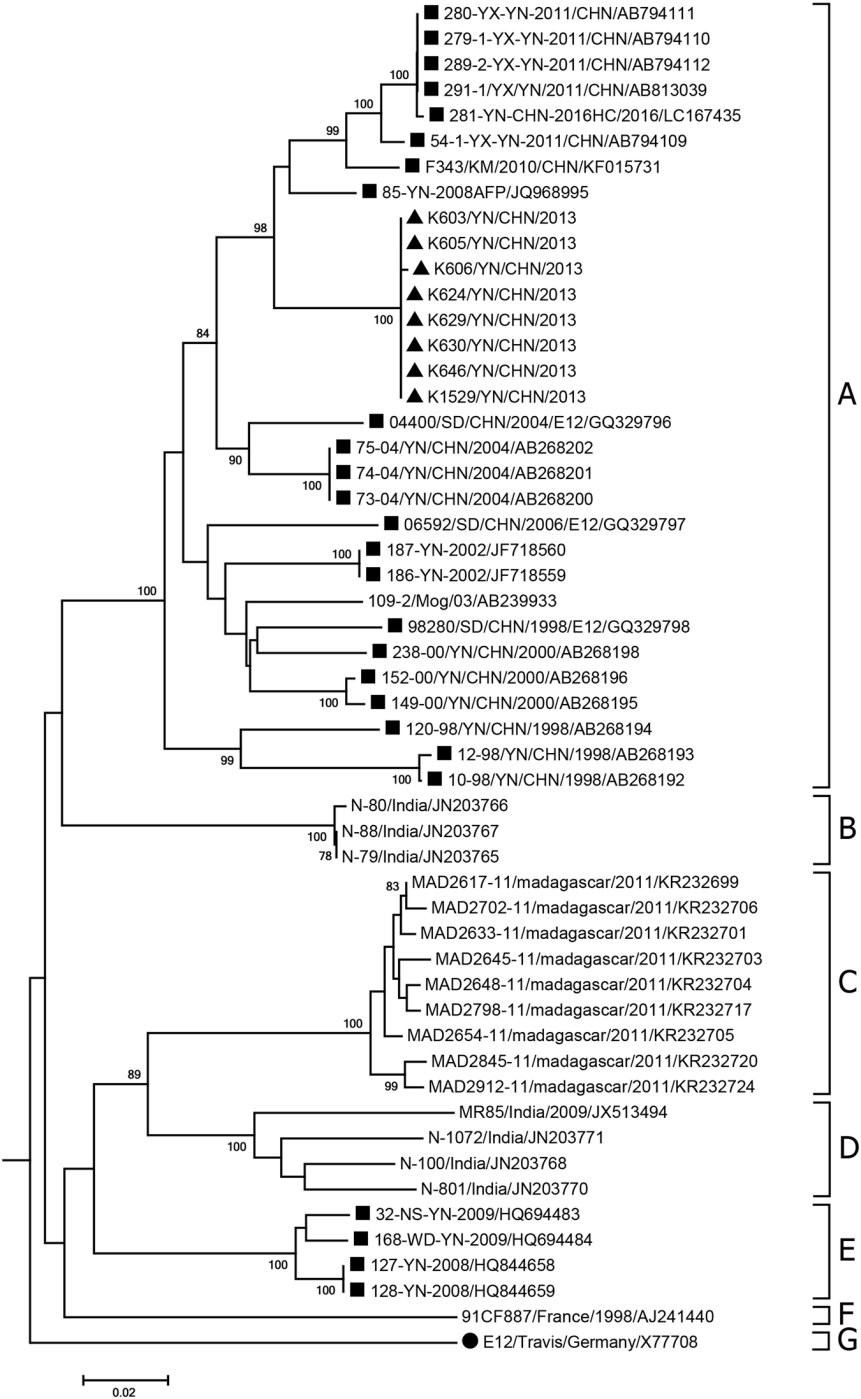
The phylogenetic tree based on partial VP1 sequences of global E-12 isolates. The triangle indicates E-12 isolates used in the present study and squares indicate other Chinese isolates. The circle indicates the prototype strain. The tree is constructed from the 657-nucleotide (positions 2617–3273) partial VP1 sequence.

Because of the lack of a complete VP1 sequence in GenBank, 53 E-12 partial VP1 sequences (including the eight Yunnan isolates from the present study) were used for sequence analysis. Sequence analysis was based on the 657-nucleotide (positions 2617–3273) partial VP1 sequences of E-12 (Fig. 1). In alignment with all 53 E-12 VP1 sequences available in GenBank, the eight isolates had 81.4–95.0% nucleotide similarity with those of the E-12 strains, and all E-12 strains were divided into seven genotypes (A-G) according to the generally accepted criterion for enterovirus genotype demarcation (the nucleotide sequence variation in the VP1 region within an enterovirus genotype should be less than 11% and the nucleotide sequence variation in the same region between enterovirus genotypes should be more than 11%)^10^. Genotype A consisted of the eight isolates from the present study, the Yunnan isolated strains, alongside three strains from the Shandong Province of China and a Mongolian strain. The other four Yunnan strains isolated between 2008 and 2009 were clustered into genotype E. Three Indian E-12 strains belonged to genotype B, and genotype D was represented by the other four Indian strains. All of the strains from genotype C were from Madagascar. The French strain and prototype strain belonged to genotypes F and G, respectively. Estimates of the average evolutionary divergence of the sequence pairs within and between genotypes are shown in Table 1. The mean nucleotide (amino acid) sequence divergence within genotype A was 9.1% (1.0%), which was below the overall mean 17.5% (1.7%) cut-off divergence value and indicated the homology between the strains isolated in the study and other Chinese E-12 strains.

**Table 1:** Mean VP1-encoding nucleotide and amino acid sequence divergence within and between the E-12 genotypes.

	A	B	C	D	E	F	G	Mean within genotype
A		1.2%	2.2%	2.2%	1.4%	2.5%	3.5%	9.1% (1.0%)
B	19.8%		1.6%	1.7%	0.5%	2.5%	2.9%	0.2% (0.3%)
C	24.7%	21.4%		2.5%	1.8%	3.5%	3.3%	1.6% (1.2%)
D	25.3%	21.0%	16.8%		1.8%	3.7%	3.7%	8.2% (2.8%)
E	21.3%	16.7%	17.1%	20.3%		2.4%	3.1%	2.0% (0.7%)
F	25.7%	23.1%	23.0%	22.8%	24.9%		5.1%	—
G	26.2%	25.4%	29.4%	28.2%	25.2%	28.6%		—

The data in the lower left corner were for nucleotide divergence analysis and the upper right corner were for amino acid divergence analysis. The data shown in the right column of the table were the mean nucleotide (amino acid) sequence divergence within each genotype.

### Complete genome analysis

The complete genome sequences of three strains named K624/YN/CHN/2013, K605/YN/CHN/2013, and K1529/YN/CHN/2013 (abbreviated as K624, K605, and K1529, respectively) isolated from Yunnan Province in 2013 were determined. The three isolates were chosen from the east, north and south of Kunming, respectively. The complete genome nucleotide sequence and amino acid similarities among the three isolates were 99.7% and 99.1–99.5%, respectively. In addition, the complete VP1 nucleotide sequence and amino acid similarities among the eight Yunnan strains were 98.4–100% and 96.6–100%, respectively. Thus, we did not determine the sequence of the 5 remaining isolates in the other genomic regions and considered the five remaining isolates to be very similar to the three fully sequenced isolates.

The genome sequences among these three strains were found to be 7,417–7,424 nucleotides in length and included a single ORF of 6,579 nucleotides encoding a single polypeptide of 2,193 amino acids. The sequences were flanked by a 5′-UTR of 743 nucleotides and a 3′-UTR of 92–99 nucleotides. The complete genome nucleotide sequence and amino acid similarities among the three Yunnan isolates were 99.7% and 99.1–99.5%, respectively. Since the complete genome nucleotide homology of these three strains was as high as 99.7%, one of them (K624) was selected for detailed analysis.

The full-length genome of the K624 strain contains 7,424 nucleotides including a 5′-UTR of 743 nucleotides followed by an ORF that encoded a polyprotein of 2,193 amino acids and a 3′-UTR of 99 nucleotides. Nucleotide and deduced amino acid sequence identities between strain K624 and echovirus prototype strains are shown in Table 2. K624 had 80.9% similarity with the complete genome of the E-12 prototype strain and the deduced amino acid sequence had 96.8% similarity.

**Table 2:** The nucleotide and amino acid identity between prototypes of E-12 and K624/YN/CHN/2013 and other prototype strains of echovirus in all of the sequenced genomic regions.

Genomic vregion	Prototype of E12		Other prototype strains of echovirus	
	%Nucleotide Identity	%Amino acid identity	%Nucleotide identity	%Amino acid Identity
5′UTR	81.2		80.2–88.0	
VP4	81.6	98.6	69.9–81.6	82.6–100.0
VP2	82.0	98.1	68.1–73.4	77.0–86.2
VP3	79.1	97.1	65.0–74.1	71.0–88.7
VP1	80.6	96.3	60.7–79.1	60.6–83.2
2 A	81.5	97.5	76.2–84.0	92.6–99.2
2B	86.1	98.0	77.1–81.9	94.9–99.0
2 C	81.2	96.4	78.7–82.5	95.4–97.9
3 A	77.4	95.6	74.5–83.1	93.3–97.8
3B	72.7	95.5	73.8–84.8	90.9–95.5
3 C	78.7	97.3	75.7–79.6	95.6–97.3
3D	79.9	96.0	77.4–81.0	95.2–97.2
3′UTR	50.0		77.9–93.8	
genome	80.9	96.8	74.7–80.8	86.4–92.3

The K624 strain shares 80.8%, 82.3%, and 79.1% nucleotide identity with the *P1*, *P2*, and *P3* coding regions of the E-12 prototype strain, respectively, and 67.7–72.7%, 79.3–82.4%, and 77.6–80.2% nucleotide identity with the *P1*, *P2*, and *P3* regions of the other E prototype strains, respectively.

### Phylogenetic analysis of *P1*, *P2*, and *P3* coding regions

**Figure 2:**
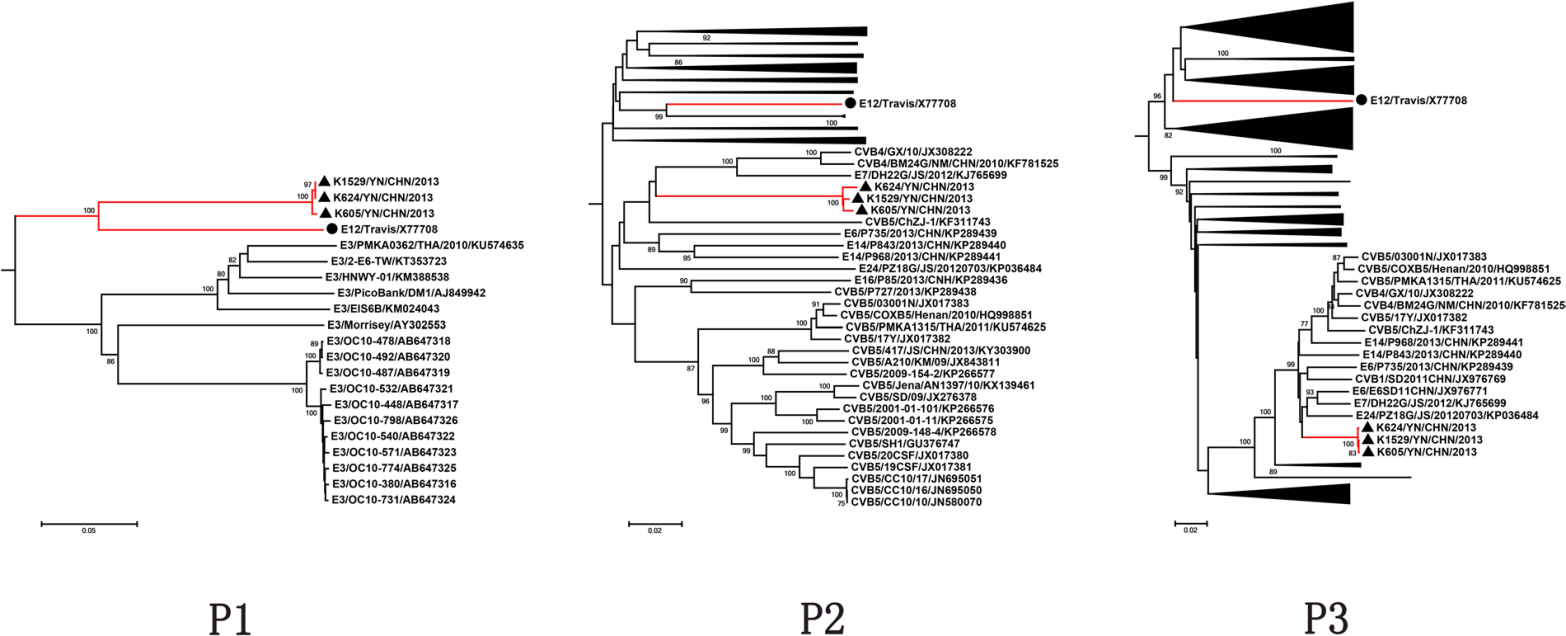
Phylogenetic relationships based on the *P1*, *P2*, and *P3* coding sequences of 381 EV-B strains. Three E-12 strains and 378 EV-B strains were analysed by nucleotide sequence alignment using the neighbour-joining algorithms implemented in the MEGA 6.06 program. The numbers at the nodes indicate bootstrap support for that node (percentage of 1,000 bootstrap replicates). The scale bars represent the genetic distance. Only high bootstrap values (>75%) are shown. ▲ indicates the strain isolated in this investigation, and ● indicates the prototype strain. The red nodes denote the other E-12 strains. The complete trees are available in Supplementary Fig. S1.

Phylogenetic analysis was based on the *P1*, *P2*, and *P3* region of the three Yunnan isolates and all of the available EV-B strains (Fig. 2). In the *P1* capsid coding region, three Yunnan isolates were clustered together with the E-12 prototype strain Travis (X77708), which is in consonance with the molecular typing results. In the *P2* region, the Yunnan isolates did not form a lineage with the E-12 prototype strain and grouped together with other EV-B serotypes such as E-7 strain DH22G/JS/2012 and two CVB4 strains (CVB4/GX/10 and CVB4/BM24G/NM/CHN/20100). Among these, the nucleotide sequence of the *P2* genomic region was the most homologous (85.5%) to the corresponding sequence of the E-7 strain DH22G/JS/2012. However, in the *P3* region, the Yunnan isolates did not form a lineage with the E-12 prototype strain and grouped together with other EV-B serotypes, especially the E-6 strain E6SD11CHN, which was the most homologous (94.0%). From the above results, this suggests that one or more putative recombination events had occurred between the E-12 Yunnan isolates and other EV-B types.

### Recombination analysis

BLAST was used to select the available sequences with high homology to K624 (Table 3). The *P2* and *P3* regions of strain K624 had the highest identity with E6SD11CHN, whereas the Yunnan strain had the highest identities (94%-100%) with the CV-B4 strain CVB4/GX/10, CV-B5 strain CVB5/PMKA1315/THA/2011, and E-6 strain E6SD11CHN in the *3A*, *3B*, *3C* and *3D* coding regions of *P3*, respectively. Among these, the nucleotide sequence of the *3B* genomic region was consistent with the corresponding sequence of CVB5/PMKA1315/THA/2011 (100%). In addition, the Yunnan strain had the highest identity (97% and 93%) with CV-B5 strain 19CSF and EV-79 strain NH95-0601 in the *3′*-UTR and *5′*-UTR regions, respectively.

**Table 3:** The highest similarity of the nucleotide sequences of enteroviruses in all of the sequenced genomic regions of the K624/YN/CHN/2013 strain using BLAST online.

Genomic region	K624/YN/CHN/2013				
	Type	Strain	%Nucleotide identity	Accession number	Disease
5′UTR	EV-79	NH95–0601	93	AB426610	—
VP4	CV-B5	China/GD237/2009	94	HQ005460	Hand, foot and mouth disease
VP2	CV-B5	China/GD237/2009	94	HQ005460	Hand, foot and mouth disease
VP3	CV-A9	EBMSV0003/THA/2010	74	KU574638	Respiratory infections
VP1	E-12	281-YN-CHN-2016HC	92	LC167435	Healthy child
2A	CV-B4	CB4 Cph1	81	KC558559	—
2B	E-30	KM/A363/09	85	KF878942	—
2C	E-6	SD11CHN	89	JX976771	Hand, foot and mouth disease
3A	CV-B4	GX/10	95	JX308222	Hand, foot and mouth disease
3B	CV-B5	PMKA1315/THA/2011	100	KU574625	Respiratory infections
3C	CV-B4	GX/10	96	JX308222	Hand, foot and mouth disease
3D	E-6	SD11CHN	94	JX976771	Hand, foot and mouth disease
3′UTR	CV-B5	19CSF	97	JX017381	Viral encephalitis
P1	E-12	281-YN-CHN-2016HC	92	LC167435	From healthy children
P2	E-6	SD11CHN	85	JX976771	Hand, foot and mouth disease
P3	E-6	SD11CHN	94	JX976771	Hand, foot and mouth disease

**Figure 3:**
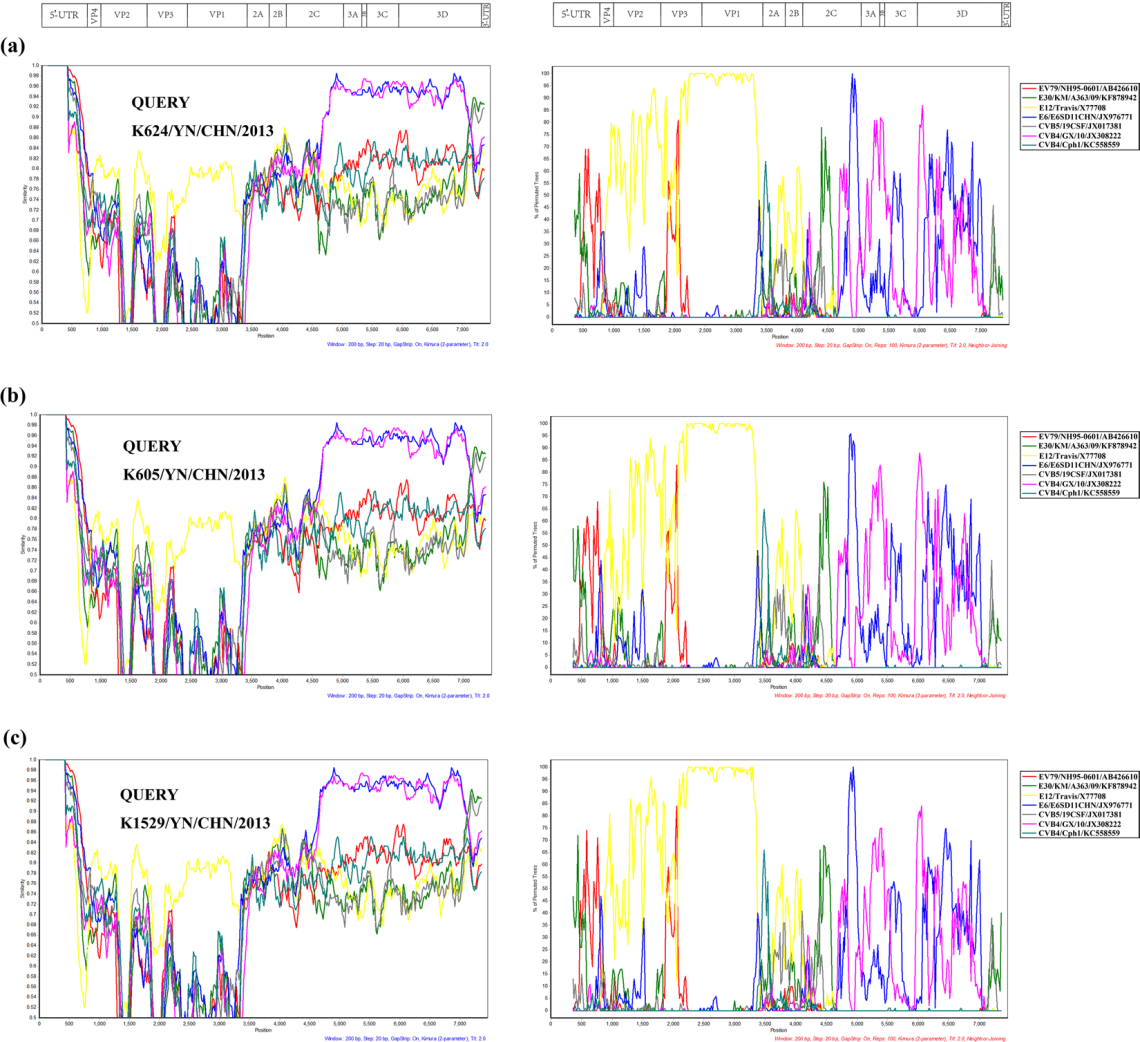
Similarity plots and bootscanning analysis of the three Chinese E-12 strains with closely related strains. The analyses were conducted via Simplot 3.5.1 using a sliding window of 200 nt moving in 20-nt steps. The genome of strain K624/YN/CHN/2013 (**a**), K605/YN/CHN/2013 (**b**), and K1529/YN/CHN/2013 (**c**) serves as a query sequence.

The sequences with the highest homology were used to conduct the recombination analysis. The similarity plot analysis and bootscanning analysis both suggested multiple recombination events in the genomic sequence of strain K624/YN/CHN/2013 (Fig. 3). In the *P1* coding region, strain K624/YN/CHN/2013 had the highest identity with the E-12 prototype strain. Whereas in the *2A* to *2C* coding regions, it was most closely related to the CV-B4 strain Cph1, E-30 strain KM/A363/09 and E-6 strain E6SD11CHN. In the *3A* to *3D* coding regions, it was most homologous with E-6 strain SD11/CHN and CV-B4 strain GX/10, respectively.

## Discussion

Following the eradication of polio, diseases caused by non-polio enteroviruses (NPEVs) have been recognized as a major public health concern in Asian countries. Among EVs, polioviruses (PVs) are the main cause of AFP. Although PV has been eradicated in China due to an intensive oral polio vaccination (OPV) program, non-polio AFP cases have been reported. NPEV genotypes associated with AFP had been reported as E-11, E-12, EV-76, E-20, E-7, E-3, E-9, CV-B1 and CV-B3^11,12^. However, most of them were collected from stool samples of AFP patients (EV-A, B, C and even D). This does not mean that these NPEVs were the causative agents of AFP. Other samples from the CNS, serum and other sites of lesions of the patients are required to elucidate the relationship.

Yunnan is not only a frontier province but also a tourist province. The population of Yunnan frequently exchanges with neighbouring Southeast or South Asian countries where AFP cases are often reported. Thus, to date, all Yunnan E-12 strains in the GenBank database accounted for the majority (41.7%) of the total isolates. In the study, all of the Yunnan strains were isolated from the stool samples of healthy populations. However, E-12 could also be isolated from the stools of patients with AFP, HFMD and diarrhoea^13–15^. Thus, the pathogenic potential of the Yunnan E-12 strains needs additional study. Furthermore, an epidemiological investigation of the association between E-12 and its related diseases needs to be conducted.

Except for four strains, the Yunnan E-12 isolates from the present study clustered with the other E-12 strains isolated from the Yunnan Province of China and these strains had the highest identity with each other, which indicated that the Yunnan strains had existed for a long time.

Recombination in enteroviruses has been reported as a major mechanism participating in the evolution of these viruses, especially EV-B^16,17^. We performed a preliminary screen with BLAST for strains that possessed a high similarity with the E-12 strains in different coding regions. Then, we proposed recombination events in different coding regions that were observed between the Yunnan isolates and the E-6 strain E6SD11CHN, CV-B4 strains (CVB4/GX/10 and CVB4/CB4 Cph1), EV-79 strain NH95-0601, CV-B5 strains (19CSF, CVB5/China/GD237/2009, and CVB5/PMKA1315/THA/2011), CV-A9 strain EBMSV0003/THA/2010, E-30 strain KM/A363/09, and E-12 strain 281-YN-CHN-2016HC. The results indicate that all of these viruses probably shared common ancestors that provided some non-structural genomic regions through recombination. Among them, the CV-A9 strain EBMSV0003/THA/2010 and the CV-B5 strain CVB5/PMKA1315/THA/2011 were isolated from patients with respiratory infections, respectively^18^; E6SD11CHN, CVB4/GX/10, and CVB5/China/GD237/2009 were isolated from patients with HFMD^19–21^; the CV-B5 strain 19CSF was isolated from patients with aseptic meningitis^22^. HFMD, which was first reported in New Zealand in 1957, is a common infectious disease mainly occurring in children under 5 years of age. Since 2008, HFMD has become a pressing issue for public health in China. It was incorporated into the C-class infectious diseases and has been ranked as one of the infectious diseases with the highest incidence in China for nearly 10 years. EVs are the causative agent of HFMD. CV-A16, EV-A71, and CV-A6 have been identified as the most frequent pathogens of HFMD, and other EVs, such as CV-B1, CV-B2, CV-B3, CV-B4 and CV-B5, can also cause HFMD^23–25^. In addition, although the surveillance system of aseptic meningitis is very limited, many studies have shown that EVs were the predominant pathogen of aseptic meningitis, and EVs associated with aseptic meningitis outbreaks often focus on EV-B serotypes such as E-30, E-6, CV-A9, CV-B3, and CV-B5^26–29^. The causative agents for HFMD and aseptic meningitis often overlap. In addition, multi-serotypes were not only co-circulated and co-infected during HFMD outbreaks and sporadic cases or asymptomatic infections but also during cases of aseptic meningitis, especially EV-B strains^30^, and the epidemiologic feature may provide ample opportunity for the virus to mingle and recombine. Thus, we suggest that Yunnan strains co-circulated with other EV-B strains and underwent extensive genetic exchanges with a few of the EV-B strains.

In conclusion, this is the first report on the entire genome of E-12 in China. Sequence analysis indicates that the Yunnan E-12 strains have a high genetic diversity compared with the other E-12 strains and intertypic recombination has occurred in the non-structural regions. E-12 strains were reported in many countries, such as the Central African Republic, Cameroon, India, Nigeria, Romania, Pakistan, and the USA. Therefore, E-12 is probably widespread (worldwide), and multiple recombination events may drive the evolution of E-12. Although these Yunnan E-12 strains were isolated from healthy children, its pathogenic potential cannot be excluded and its pathogenicity needs further research and study. Epidemiological surveillance should continue to evaluate the association between E-12 and its related diseases.

## Materials and Methods

### Ethics statement

Parents and/or legal guardians of the participates in this study gave written informed consent. The protocol was in accordance with the Helsinki Declaration and was approved by the Institutional Ethics Boards of the Institute of Medical Biology, Chinese Academy of Medical Sciences & Peking Union Medical College. The human materials in the study were stool samples collected from eight healthy children aged 3–6 years.

### Sample collection and virus isolation

A total of 96 stool samples were collected from three kindergartens from June to August in 2013 in Kunming, China. The three kindergartens are located in the east, north and south of Kunming. The children were asymptomatic and did not present a fever, cough, or rash and did not have contact with patients recently infected by EVs in the 2 weeks before sample collection. The children’s ages ranged from 3 to 6 years^31^. The cell line, KMB17, was used to isolate viruses from the stool specimens according to the standard procedures^32^. The samples that induced a cytopathic effect (CPE) were considered positive. All of the positive isolates were stored at −80 °C.

### VP1 RT-PCR, sequencing, and typing

Viral RNA was extracted from the cell culture supernatants with a QIAamp Viral RNA Mini Kit (Qiagen, Valencia, CA, USA) in accordance with the manufacturer’s instructions. The enterovirus universal primer pairs 222 and 224 were used to amplify the partial VP1 gene sequence^33^ using a PrimeScript^TM^ One-Step RT-PCR Kit Ver.2 (Takara, Dalian, China). The positive PCR products were sequenced using an ABI 3730XL automatic sequencer (Applied Biosystems, Foster City, CA, USA) at the Tsingke Sequencing Company (Kunming, China).

The partial VP1 nucleotide sequence (nucleotide position 2617–3273) was analysed using the Enterovirus Genotyping Tool for serotyping^34^. Virus strains showing >75% nucleotide sequence identity with a known enterovirus serotype were designated the relative serotype of EVs. Estimates of average evolutionary divergence of the sequence pairs within and between E-12 genotypes were performed using MEGA6 (Table 1). The analyses were conducted using the Tamura-Nei model. The rate variation among the sites was modelled with a gamma distribution (shape parameter = 1).

### Whole genomic sequencing

Primers for amplifying the complete genome were designed using the published sequence of the Travis strain and internal primers were designed for the ‘primer walking’ strategy as previously reported^35^. The positive PCR products were sequenced using an ABI 3730XL automatic sequencer (Applied Biosystems, Foster City, CA, USA) at the Tsingke Sequencing Company. The primers for PCR amplification and sequencing are listed in Table 4. The 5′ extremity of the three genomes was not sequenced by RACE RT-PCR or circularization.

**Table 4:** Primers for complete genome amplification and sequencing.

Primer	Sequence (5′ → 3′)	Nucleotide position	Orientation
224	GCIATGYTIGGIACICAYRT	1977–1996	Forward
222	CICCIGGIGGIAYRWACAT	2969–2951	Reverse
E201F	TTAAAACAGCCTGTGGGTTG	1–20	Forward
E122R	ACACACACGCTGCGCGGCAG	2671–2652	Reverse
E123F	TTGGCGCATCAATACTCGAC	2641–2660	Forward
E208R	ACCGAATGCGGAGAATTTAC	7450–7431	Reverse
E123f	GACGTAACCACCACACGC	3256–3273	Forward
E122r	GGGGCTTTGTTCATTCAC	3135–3118	Reverse
E128f	TCATGACACCAGCAGACA	6985–7002	Forward
E125r	ATCATACCCACTGTAATC	5883–5866	Reverse
E124f	CAACAGACTCAAACAGCT	4185–4202	Forward
E122r1	GTCTCCTGGTACCACTTG	2586–2569	Reverse
E125f	CCACCCGTGTATAGAGAG	5051–5068	Forward
E125r	TTATTCCCTTCAAAGA	6025–6010	Reverse
E121f	GTTACCATATAGCTATT	566–582	Forward
E122rrr	TCACCTCCCCTGGTAT	1833–1813	Reverse
E121f	GTTACCATATAGCTATT	566–582	Forward
E123r	CCTGGGCCTTCAAAGCT	3152–3136	Reverse
E121r	ACCAATTAGCTCAATA	399–384	Reverse
E122rr	GCTATCTCCATTAAGTT	1827–1818	Reverse

### Sequence analysis and recombination analysis

BioEdit (version 7.2.3) was used for nucleotide and amino acid sequence alignment. The phylogeny of the E-12 strains and other members of the species EV-B strains was conducted via MEGA (version 7.0)^36^ and generated by the neighbour-joining method with a Kimura two-parameter model. Bootstrapping was performed with 1000 duplicates and the bootstrap values greater than 75% were considered statistically significant for grouping. The Simplot 3.5.1 software, which was programmed with a 200-nt window moving in 20-nt steps and a Jukes-Cantor correction^37^, was used for the similarity plot and bootscanning analysis. The highest similarity of the nucleotide sequences of enteroviruses in all of the sequenced genomic regions of the K624/YN/CHN/2103 strain using BLAST online is shown in Table 3.

### Nucleotide sequence accession number

The accession numbers of the complete VP1 nucleotide sequences and full-length genome sequences of the E-12 strains identified in this study were as follows: MF083144-MF083151 and MF083152-MF083154, respectively.

## Electronic supplementary material

Supplementary Information
