# Supplementary material for: Molecular characterization of echovirus 12 strains isolated from healthy children in China
Source: Sci Rep. 2018 Aug 6;8:11716. doi: 10.1038/s41598-018-30250-x (PMC6078983; doi:10.1038/s41598-018-30250-x)
Supplement: Supplementary file 1 — Supplementary Information [file 41598_2018_30250_MOESM1_ESM.pdf]

Supplementary Information

Molecular characterization of echovirus 12 strains isolated from healthy children in China

Hongbo Liu[1,2*], Jie Zhang[1,2*], Yilin Zhao[1,2],Haihao Zhang[1,2] , Keqin Lin[1,2], Hao Sun[1,2], Xiaoqin Huang[1,2], Zhaoqing Yang[1,2], Shaohui Ma[1,2]

[1] Institute of Medical Biology, Chinese Academy of Medical Sciences, and Peking Union Medical College, Kunming 650118, PR China. [2] Yunnan Key Laboratory of Vaccine Research Development on Severe Infectious Disease, Kunming 650118, PR China.

*Hongbo Liu and Jie Zhang have contributed equally to this study. Correspondence and requests for materials should be addressed to Z.Y. (email: zyang@imbcams.com.cn) or S.M. (email: shaohuima70@hotmail.com)

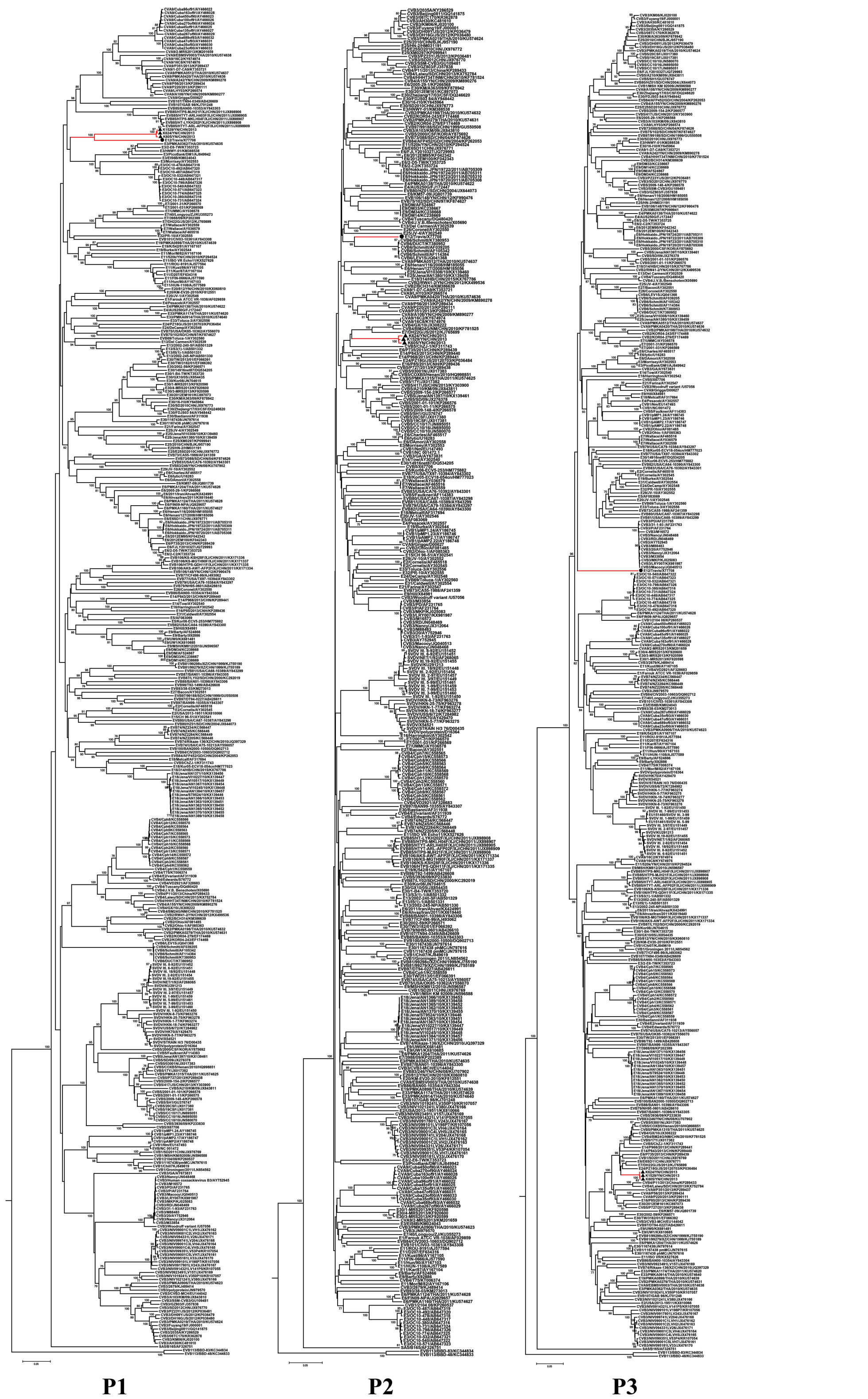

P1 P2 P3

Supplementary Figure S1. Complete Phylogenetic tree based on the *P1*, *P2*, and *P3* coding sequences of 381 EV-B strains, using the neighbor joining algorithms implemented in the MEGA 6.06 program. Numbers at the nodes indicate bootstrap support for that node (percentage of 1,000 bootstrap replicates). Only high bootstrap values (>75%) are shown. ▲ indicates the strain isolated in this investigation, and ● indicates the E-12 prototype strain. The red nodes denote the other E-12 strains.
